# Deconstructing Apathy in Parkinson's Disease: Challenges in Isolating Core Components of Apathy From Depression, Anxiety, and Fatigue

**DOI:** 10.3389/fneur.2021.720921

**Published:** 2021-08-26

**Authors:** Christian Ineichen, Heide Baumann-Vogel

**Affiliations:** Department of Neurology, University Hospital Zurich, University of Zurich, Zurich, Switzerland

**Keywords:** Parkinson's disease, apathy, depression, fatigue, anxiety, confirmatory factor analysis, nosology

## Abstract

Apathy, fatigue and depression are amongst the most debilitating non-motor syndromes of Parkinson's disease (PD). The aim of this study was to examine the prevalence of apathy, depression, anxiety and fatigue and whether these syndromes are separable in PD. A total of 337 patients were examined using the Unified Parkinson's Disease Rating Scale (UPDRS part III), the Apathy Evaluation Scale, the Hospital Anxiety and Depression Scale and the Fatigue Severity Scale. Using standard cutoff criteria, the prevalence rates of significant apathy, mild-to-severe depression, mild-to-severe anxiety and severe fatigue were 23.7, 13.4, 15.4, and 17.8%, respectively. Next, confirmatory factor analysis was employed of items from these three clinical scales. A priori hypothesis testing including four different factors (reduced motivation/interest, physical fatigue, reduced pleasure, anxiety) was performed. The factor analysis revealed strong fit statistics for the model with χ^2^ (57, *N* = 377) = 58.9, *p* = 0.41, CMIN/DF = 1,034, NFI = 0.977, CFI = 0.999, IFI = 0.999, RFI = 0.968, and TLI = 0.999. The RMSEA was 0.01, and the standardized RMR was 0.027. These results support the hypothesis that apathy, fatigue, depression and anxiety represent prevalent syndromes that can be separated in Parkinson's disease and that apathy is not just a subcomponent of depression or fatigue. The results of this study may contribute to a clearer diagnostic process for apathy, fatigue and depression and may aid in patient care.

## Introduction

Apathy is a multifaceted syndrome related to reduced goal directed behavior associated with emotional, cognitive, and behavioral components that may result in such diverse states as indifference, reduced or lack of concern, initiative, and decision making with far reaching consequences for daily living (1, 2). Awareness of the significance of apathy but also other overlapping symptoms of depression and fatigue in patients suffering from Parkinson's disease (PD) has increased recently. As part of the dopaminergic but also serotonergic degeneration, they constitute an integral part of PD and have considerable impact on disability and quality of life (3)—with the latter representing a major burden not only for the individual patient but also for the social environment. In PD, apathy is associated with poor response to motor symptom treatment, increased risk for developing dementia and difficulties to make daily life decisions. Moreover, there is evidence that apathy is a direct consequence of the pathology itself rather than a consequence of psychological distress based on motor disability (4). Hence, apathy can be found both in early and advanced stages of PD (5, 6). The mean prevalence of apathy in PD has been estimated to be 40%, which exceeds the rates found in other neurodegenerative disorders (7). However, there is a large heterogeneity in assessment tools and evidence that a substantial number of studies have used non-recommended instruments which makes both diagnosis and, as a direct consequence, prevalence estimates of apathy difficult (8). Nowadays, apathy is considered to represent a syndrome which is characterized by loss or reduction of motivation and at least one of decreased goal-directed behavior, goal-directed cognition and/or emotional reactivity (9, 10). However, there is disagreement about the multifaceted subcomponents that may or may not account for apathy. For example, in many publications, the terms apathy, fatigue and anhedonia are used without clear conceptual demarcation. More precisely, as Husain and Roiser (7) have outlined, there is a considerable debate as to what extend the symptoms of anergia relating to lack of energy and fatigue, the tiredness following physical or mental activity, are dissociable from apathy. The nosological position of apathy is further complicated by the fact that abulia (i.e., inability to perform selfdirected, purposeful activities) and avolition (i.e., reduced spontaneous verbal, motor, cognitive and emotional behaviors), both associated with action initiation, constitute two further symptoms which delineation in the context of apathy is unclear. In addition, amotivation in apathy is multifaceted and extends to behavioral, cognitive, emotional but also social domains [for social apathy, see (11)]. It can therefore sometimes be associated with social withdrawal and other neuropsychological phenomena. Regarding the unclear interrelation of apathy and affect, it has been argued that it is apathy that represents the underlying source of amotivation independent of a potential emotional challenge (1, 12). Therefore, this and other more recent investigations speculate about a potentially important dissociation between an affective component and apathy (13). Finally, the relation of apathy and amotivation in depression and the amotivational component of anhedonia (a further unclear conceptual mixture of the clinical features of loss of interest and pleasure), shows also considerable theoretical overlap. Studies in PD patients support a strong interrelation between apathy and anhedonia insofar as significant positive correlations between scores on apathy and anhedonia scales have been shown (14, 15). It is however a question whether anhedonia in PD—at least in terms of inability to experience pleasure—can be separated from apathy (13). While there is evidence that anhedonia responds to dopaminergic treatment (15) due to parkinsonian mesocortical- and mesolimbic denervation, there are variable and inconclusive views on the role of dopaminergic and serotonergic alterations in apathy and their potential targets for treatment (16–18)—which may also point to the outlined lack of conceptual clarity. Albeit a mutual incompatibility of apathy (roughly characterized as a state of hypoarousal) and anxiety (roughly characterized as a state of hyperarousal) could be inferred on first sight, the relationship of apathy and anxiety is more complex. This is corroborated by the fact that they often co-exist in PD and Alzheimer disease (17, 19).

So far, success in treating apathy in PD is mainly a consequence of treating other neuropsychiatric symptoms such as improvement in apathy due to cholinesterase inhibitors as a secondary outcome to improvements in cognitive impairment or the treatment of associated depression (20). Without a doubt, the lack of consensus regarding the definition of apathy limits the diagnostic approach taken to identify apathy in PD which may lead to misdiagnosis and pseudo-syndromes (21).

More information on whether apathy is a subcomponent of fatigue and depression in PD and therefore a better separation of these symptoms would have significant implications for studying their underlying neural mechanisms in aetiopathogenesis as well as for differential diagnosis and treatment. Therefore, using confirmatory factor analysis (CFA) of individual items from three commonly used clinical rating scales, the aim of this study was to investigate the hypothesis that apathy, fatigue and depression are dissociable in PD. Because of the large conceptual overlap in these clinical scales, conducting CFA might provide insights into relevant item-clusters that are clearly separable from each other. Based on clinical (treatment) experience and theoretical considerations [e.g., see (1, 2, 7, 9, 13, 15, 22–24)], we aimed at testing a model consisting of the following four factors A1: pure apathy (amotivation; free of any hedonic component) potentially distinguishable from A1 pot: loss of interest (conceptualized as reduced goal-directed cognition), B1: physical fatigue and disease burden (effort and stamina; in contrast to mental fatigue and fatigability), C1: loss of positive mood (as the pleasure-component of anhedonia) and D1: anxiety (as a separate cluster on the other side of the spectrum relative to C1) (see Supplementary Table 1 for a list of the individual items and their allocation to the hypothesized clusters).

## Materials and Methods

### Subjects

Data records from 337 patients (mean age 69.3 years, SD: 10.2, 38.3% women, mean disease duration 13 years, mean Hoehn and Yahr stage: 2.4, SD: 0.7) suffering from PD and recruited from the university hospital in Zurich were retrospectively analyzed. The inclusion criteria were age older than 18 years, diagnosed PD according to established criteria (25), complete clinical assessment, and informed consent to participate. Patients with severe unstable psychiatric disorders (e.g., psychotic or manic episode) were excluded. Because cognitive deficits are common in PD, we assessed the frequency of mild cognitive impairment or dementia using data from the Montreal Cognitive Assessment (MoCA). With a frequency of 5% (17 patients), the number of patients suffering from mild cognitive impairment was low. Demographic characteristics, disease duration, Levodopa Equivalent Daily Dose (LEDD), total dose of dopamine agonists (total DA) and severity of motor symptoms assessed by the Unified Parkinson's Disease Rating Scale (UPDRS, part III) during the medication ON state were recorded (Table 1). All participants gave their written informed consent to using their anonymized data for this retrospective analysis, which was approved by the local ethics committee.

**Table 1:** Patient characteristics.

**Characteristic**		**PD patients (*N* = 337)**
Age	X	69.3
	SD	10.2
Female	%	38.3
Disease duration	X	13 years
Hoehn and Yahr stage	X	2.4
	SD	0.7
Frequency of mild cognitive impairment	%	5.0
Levodopa medication	%	97.3
Antidepressant medication	%	49.0
Dopamine agonist medication	%	65.3
Levodopa equivalent dose (mg)	X	696.8
	SD	782.6
Dopamine Agonist dose (mg)	X	63.4
	SD	84.3
Motor score (UPDRS-III)	X	20.8
	SD	10.2
Apathy Scale (AES)	X	33.5
	SD	8.0
Depression Subscale (HADS)	X	5.7
	SD	3.5
Anxiety Subscale (HADS)	X	6.2
	SD	3.5
Fatigue Scale (FSS)	X	3.8
	SD	1.4

### Clinical Assessment

Apathy was quantified using the German version of the Apathy Evaluation Scale (26) which consists of 18 items each scored on a (self-report) 4-point Likert-scale (total AES score range 18–72). Scores for items 6, 10, and 11 were all reverse-coded prior to calculating individual summation scores [see (27), for more information on psychometric properties of the scale]. The AES represents one of the most clinically used rating scales for the measurement of apathy. Depression was assessed using the German version of the Hospital Anxiety and Depression Scale [HADS; (28)] which allows for the separate assessment of depression and anxiety. It consists of 14 items each scored on a self-report 4-point Likert scale, 7 of which assessing anxiety (A-HADS in the following) whereas the other 7 items assessing depression (D-HADS in the following) [see (29) for more information on psychometric properties of the scale]. Fatigue was assessed using the Fatigue Severity Scale [FSS; for a validity analysis see (30); German version (31)] consisting of 9 items each scored on a 7-point Likert scale. First and foremost, it measures physical fatigue but also mental fatigue and disease burden are measured. Moreover, the FSS is the only “recommended” fatigue scale for screening and quantifying severity of PD subjective fatigue as assessed by the MDS Task Force (32). Motor impairment was measured using the Unified Parkinson's Disease Rating Scale (UPDRS, Part III) consisting of the motor examination and including 27 items in total.

### Statistical Analysis

We conducted descriptive statistical analysis using Python as well as SPSS Statistics 25.0 (IBM), complemented by inferential statistics including spearman correlational analysis and confirmatory factor analysis (CFA). Significance was accepted at a *p* < 0.05 level. Confirmatory factor analysis was conducted with the statistical software AMOS 25.0, using maximum likelihood estimation. Before performing statistical analyses, the dataset was screened for missing data and outliers. Before executing CFA, assumptions for conducting CFA were tested: Testing for multinormality (Mahalanobis critical value (28 cases excluded) as well as testing for skewness and kurtosis) revealed a fairly normal distribution of indicators of latent factors which were consistent with the recommendations of Kline (33). Testing for multicollinearity, the collinearity statistics revealed no violation. No violation was observed when testing for heteroscedastic data. A-priori sample size calculation for SEM revealed a minimum sample size of 342 to detect a small-medium effect size (0.2) using 4 latent and 19 observed variables. Moreover, the appropriateness of the sample size was tested by investigating the KMO of a pseudo-exploratory factor analysis which also demonstrated that positive definiteness was not violated. In addition, it provided reliability statistics using item-correlations and Cronbach α (not shown; of note is that the AES-item “KLessConcerned” was excluded due to below acceptable inter-correlation values and its effect in reducing the overall Cronbach α). Models were checked for fit based on the following goodness-of-fit criteria: minimum fit function χ^2^, root mean square error of approximation (RMSEA), root mean square residual (RMR), normed fit index (NFI), comparative fit index (CFI), incremental fit index (IFI), relative fit index (RFI), and the Tucker-Lewis Index (TLI). Standard quality recommendations were used to determine goodness of fit. These included a ratio of χ^2^ to degrees of freedom of 2:1 or less (CMIN/DF; as the χ^2^ is particularly vulnerable to large samples), values of RMSEA below 0.05 and non-significant, standardized RMR below 0.05, and NFI, CFI, IFI, RFI, TLI above 0.95 (34). To aid model comparison the Akaike (AIC) and Bayesian Information Criterion (BIC) were also consulted. The proposed 4-factor model was tested against 1-, 2-, and 3-factor models and examined for changes in fit. In addition, a 5-factor model was tested using the potentially separable factor of loss of interest (as the cognitive, amotivational component of anhedonia).

## Results

Demographics and characteristics for all subjects are displayed in Table 1.

### Prevalance of Apathy, Depression, Anxiety, and Fatigue

**Figure 1:**
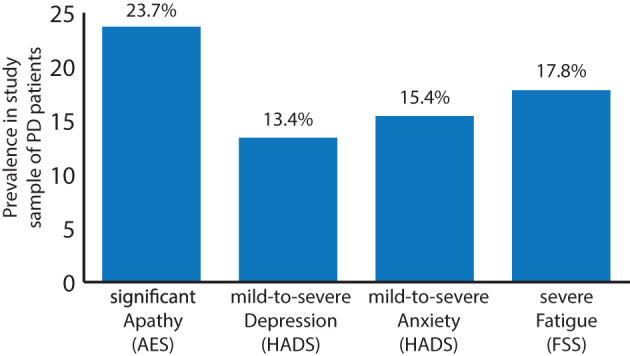
Prevalence of clinically significant apathy (AES total score ≥ 40; 23.7%), mild-to-severe depression (HADS partial score ≥ 10; 13.4%), mild-to-severe anxiety (HADS partial score ≥ 10; 15.4%), and severe fatigue [FSS total score ≥ 5; 17.8%; significant fatigue (FSS total score of ≥ 4): 40.3%; not shown] in a sample (*n* = 337) of patients suffering from PD.

First, the prevalence of apathy, depression and anxiety was assessed, using the following cutoff-scores: an AES score of 40 and above [relating to clinically significant apathy (35)] and a score of 10 and above for depression and anxiety components of the HADS, respectively [relating to mild-to-severe depression or anxiety (36)]. Eighty of the 337 patients (23.7%), showed an AES score above the cutoff value, indicating clinically meaningful apathy whilst 45 and 52 of the 337 patients (13.4 and 15.4%) had an above-cutoff value of HADS indicating mild-to-severe depression and anxiety, respectively. A total of 26 (7.7%) patients showed a clinical mixture of apathy and anxiety, 27 patients (8.0%) a combined above cutoff value for apathy and depression and 23 (6.8%) patients above cutoff values for both anxiety and depression, reflecting a substantial co-occurrence of these symptom-clusters. Finally, 16 patients (4.8%) showed above cutoff values for all three measures. For the FSS, a cutoff value of above 4 (152 patients, 40.3%) is considered significant fatigue whereas values above 5 (67 patients, 17.8%) are considered severe fatigue (31). Of the patients in this sample, 11 (3.3%) patients exceeded the relevant thresholds for apathy, depression, severe fatigue and anxiety, whereas 16 (4.8%) patients fulfilled the criteria for apathy, depression and severe fatigue and 34 (10.1%) patients for apathy and severe fatigue (Figure 1).

### Correlational Analysis

**Figure 2:**
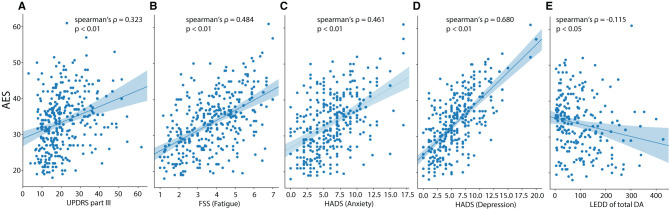
Scatterplots for apathy (AES total score) vs. **(A)** motor impairment (UPDRS-III), **(B)** fatigue (FSS), **(C)** anxiety (HADS), **(D)** depression (HADS) and **(E)** LEDD of total dopamine agonists. The regression lines are presented together with Spearman's correlation coefficient (*ρ* and the associated *p*-value).

Next, we conducted a spearman correlational analysis using the respective global or partial scores of the AES, HADS and FSS: a higher AES score (indicating greater apathy) was associated with higher motor impairment (UPDRS part III; ρ = 0.323, *p* < 0.01), higher fatigue (ρ = 0.484, *p* < 0.01) and higher anxiety- and depression scores (ρ = 0.461, *p* < 0.01, ρ = 0.680, *p* < 0.01, respectively) (Figure 2). The AES was associated with age (weak positive association, ρ = 0.156, *p* < 0.05) and the total dose of dopamine agonists (weak negative association, ρ = −0.115, *p* < 0.05). There was a weak positive association between the AES and the total levodopa equivalent dose (ρ = 0.137, *p* < 0.05).

### Confirmatory Factor Analysis

**Figure 3:**
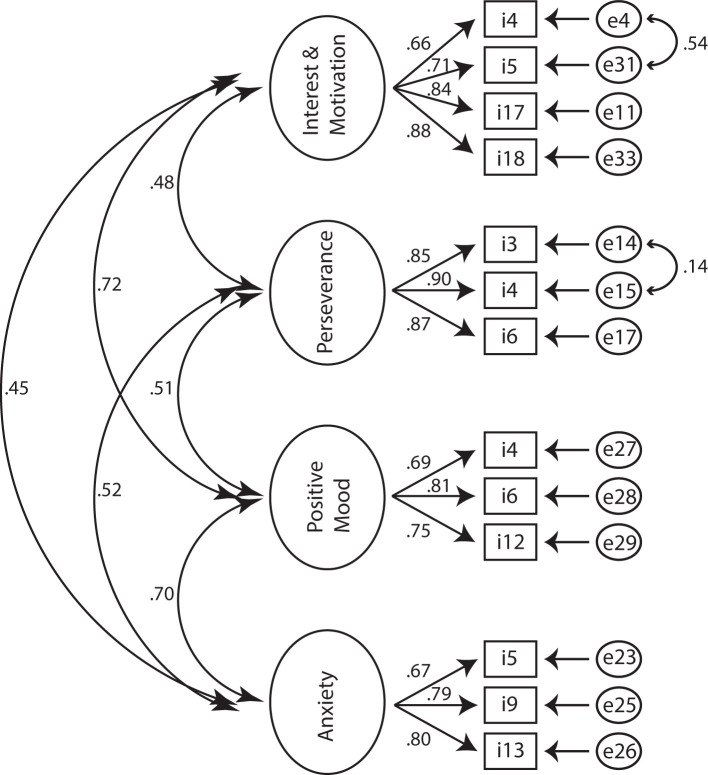
Standardized estimates for the four-factor structure. Observed indicators (items) are enclosed in rectangles, latent variables (factors) in large circles, whereas measurement errors are enclosed in ellipses.

Thirdly, CFA was used to investigate whether pure apathy including the interest domain of anhedonia (AES: items #1-11,14,16-18; FSS: items #1; HADS: items #8,10), physical fatigue (FSS: items #2,3,4,6, including disease burden: FSS: items #7-9), depression including the loss of pleasure component of anhedonia (D-HADS: items #2,4,6,12) and anxiety (A-HADS: items #3,5,9,13) measured based on AES, HADS and FSS are dissociable constructs in PD. After removing items with unexplained variance, we aimed at testing whether items cluster reliably on our a-priori factors including an “apathy” cluster (A1) including the items C_StartingThingsImportant, D_NewExperiences, E_InterestedLearning, G_Vitality, P_GettingThingsDoneDaily, Q_Initiative, and R_Motivated, another factor related to physical fatigue and disease burden (B1) and composed of the items IsMostAfffectingProblem, DutiesInhibited, TiredQuickly, ProductivityAffected, Problems, and ProductivtyInhibited, a third factor related to positive mood (or lack of positive mood as a common depressive symptom) (C1) comprising the items CanLaugh, Happy and FutureIsBright, and a final factor related to anxiety (D1) including the items Worrying, WorryingGutFeeling, Panic. The overall fit of the model to the data was: χ^2^ (146, *N* = 337) = 511.5, *p* < 0.01 (NFI = 0.891, CFI = 0.919, IFI = 0.920, RFI = 0.872, TLI = 0.905). The root mean square error of approximation (RMSEA) was 0.086 (*p* < 0.01), and the RMR was 0.0402. The overall fit in terms of the ratio of χ^2^ to degrees of freedom ratio was larger than a ratio of 2:1 (3.5:1). Modification indices as well as standardized residual covariances were examined to improve the fit of the model. This led to the removal of items yielding a further deconstruction (including the removal of items relating to disease burden) of the theoretical model and a subsequent 4-cluster model including the factors: (A) interest and motivation (B) perseverance, (C) positive mood, and (D) anxiety. Cluster (A) was composed of items A1_NewExperiences, A2_InterestedLearning, A3_Initiative, A4_Motivated, cluster (B) of items B1_TiredQuickly, B2_ProductivityAffected, B3_ProductivityInhibited, cluster (C) of items C1_CanLaugh, C2_Happy, C3_FutureIsBright, and cluster (D) was composed of items D1_Worrisome, D2_WorringGutFeeling and D3_Panic. Interestingly, modification indices suggested that there was unexplained variance among A1_NewExperiences and A2_InterestedLearning as well as among B1_TiredQuickly and B2_ProductivityAffected. Allowing the unexplained variance of these indicators to correlate, improved the model by 88.24 χ^2^ points. The final model yielded an overall fit of: χ^2^ (57, *N* = 377) = 58.9, *p* = 0.41, CMIN/DF = 1,034 (NFI = 0.977, CFI = 0.999, IFI = 0.999, RFI = 0.968, TLI = 0.999). The RMSEA was 0.01 (*p* = 0.9), and the standardized RMR was 0.027. The validity was tested according to Gaskin et al. [(37), but see also (34, 38)]. Factor loadings for cluster (A) ranged between 0.658 and 0.880 (motivation), for cluster (B) between 0.851 and 0.903 (productivity affected), for cluster (C) between 0.692 and 0.808 (happy), and for cluster (D) between 0.673 and 0.802 (panic) (Figure 3). Inter-factor correlations ranged between 0.447 (cluster pair: A–D) and 0.724 (cluster pair: A–C) (Figure 3). Neither a three-factor model including apathy, bodily fatigue and anhedonia, nor a five-factor model separating interest from factor (A) did yield a better fit (see Table 2 for model comparison). Therefore, the 4-factor model separating cluster motivation/interest (A), perseverance (B), positive thinking/mood (C), and anxiety (D) was supported.

**Table 2:** Goodness-of-Fit Indices for three models in the PD-patients sample.

**Model**	**χ** ^**2**^	**d.f**.	***p***	**χ**^**2**^**/d.f**.	**GFI**	**NFI**	**RFI**	**IFI**	**TLI**	**CFI**	**RMSEA**	**AIC**	**BIC**
M1	113.8	61	0.001	1.866	0.95	0.96	0.95	0.98	0.98	0.98	0.051	173.8	288.4
M2	58.9	57	0.4	1.034	0.98	0.98	0.97	0.99	0.99	0.99	0.010	152.9	256.8
M3	126.2	80	0.001	1.577	0.95	0.96	0.95	0.99	0.98	0.99	0.041	206.2	359.0

*χ^2^/d.f., relative chi-square; NFI, normed fit index; RFI, relative fit index; IFI, incremental fit index; TLI, Tucker-Lewis Index; CFI, comparative fit index; RMSEA, root meant square error of approximation; AIC, Akaike Information Criterion; BIC, Bayesian Information Criterion; M1, three factors (13 items); M2, four factors (13 items); M3, five factors (15 items)*.

Internal validity of the four-factor model was verified by principal component factor analysis. Construct validity is confirmed through structural equation modeling (SEM) by inspecting the standardized regression coefficients in the regression of observed variables on latent variables. As shown in Table 3, all the items had *t*-values above 2.58 (*p* < 0.01) which means that the four-factor model has strong constructs [i.e., the measured variables or factors strongly represent the underlying constructs (39)].

**Table 3:** Parameter estimates, error terms, and *t*-values for the four-factor model.

**Factor**	**Items**	**Standardized factor loadings**	**Error term**	***t*-values** ^*^
Motivation/Interest	i4: NewExperiences	0.658	0.056	12.98
	i5: InterestedLearning	0.712	0.055	14.44
	i17: Initiative	0.838	0.052	17.80
	i18: Motivated	0.880	^*†*^	^*†*^
Perseverance	i3: TiredQuickly	0.851	0.066	13.51
	i4: ProductivityAffected	0.903	0.065	14.31
	i6: ProductivityInhibited	0.866	^*†*^	^*†*^
Positive mood	i4: CanLaugh	0.692	0.068	11.84
	i6: Happy	0.808	0.071	13.60
	i12: FutureIsBright	0.748	^*†*^	^*†*^
Anxiety	i5: Worrisome	0.673	0.074	11.64
	i9: WorryingGutFeeling	0.788	^*†*^	^*†*^
	i13: Panic	0.802	0.073	13.42

* *t-values >2.58 considered significant at the 0.01 level*.

† *A parameter fixed at 1.0 in the original solution*.

## Discussion

One of the main obstacles for appropriately identifying and managing apathy in PD is the lack of gold standard criteria. Accordingly, there is still disagreement concerning the construct of apathy and its relation to depression and fatigue in clinical practice, particularly in disorders such as PD in which all of these neuropsychiatric syndromes may occur concomitantly. Without a distinct differentiation and diagnosis of the clusters of symptoms within one or another construct, the effective management of apathy, depression and fatigue remains hampered. Moreover, this lack of definitional clarity represents an impediment for the validation of measurement tools leading to measurement variation and variable prevalence estimations of apathy and related syndromes in PD.

The presence of debilitating apathy negatively impacts on treatments for other interventions such as engaged participation in physiotherapy or taking advantage of the mobility gained by an improvement of motor symptoms due to optimal dopaminergic treatment or deep brain stimulation (DBS). Therefore, the assessment and accurate differential diagnosis of apathy and related syndromes remains crucial.

With the aim of crystallizing a possible core of apathy and thus contributing to a clearer diagnostic process for apathy, this study examined whether apathy, depression, anxiety and fatigue can be separated in PD.

### Prevalances of Apathy, Depression, Anxiety, and Fatigue in the Sample of PD Patients

With 23.7, 13.4, 15.4, and 17.8%, the prevalence rates of significant apathy, mild-to-severe depression and anxiety and severe fatigue as assessed by the AES, HADS and FSS are comparable to other clinical PD samples (7, 23, 40, 41)—albeit at the lower end of the spectrum. It must be noted that there are large ranges in published prevalence rates of psychiatric syndromes in PD possibly due to different measurement methods used in the various studies, different cut-off thresholds (e.g., a prevalence of 40.3% for significant fatigue is observed in the current sample) and clinical variability of study subjects. With almost ¼ of the patients showing significant apathetic symptoms, followed by severe fatigue, mild-to-severe anxiety and depression, apathy is the most frequent syndrome measured in this population of PD patients as assessed by the selected cut-off thresholds.

### Correlational Analysis

The correlational analysis identified strong positive relationships between apathy, depression, anxiety, and fatigue besides confirming that in addition, PD apathy is associated with older age (weak association in the present sample) and lower dopamine agonist use (weak association), as has been shown before [e.g., (42)]. None of LED-total dose, LEDD of total DA, disease duration, age, or sex were strongly associated with PD fatigue and depressive or anxious symptoms. The strong positive correlations between apathy, depression and fatigue may reflect the large theoretical overlap of these syndromes. Based on the hypothesized overlap of symptoms when using total scores, these correlations have to be taken with caution.

### Pure Apathy and the Construction of Homogeneous Clusters

The large degree of theoretic ambiguity in the structure of these scales limits the interpretation of the correlational results. Therefore, we aimed at testing whether items of three widely used clinical scales (AES, HADS, FSS) would cluster in four separate factors including (A) pure apathy (motivation and interest) (B) physical fatigue (C) loss of positive affect (pure anhedonia), and (D) anxiety. After removing variables with unexplained variance and consulting modification indices as well as standardized residual covariances, there were no items that clustered on single factors originating from different scales for the apathy cluster. Moreover, the model fit was highest when a hypothesized separate cluster including loss of interest was merged with the amotivation-factor.

The final model demonstrating strong model fit characteristics was composed of the following final factors:

**A)** (loss of) interest and motivation
**B)** perseverance
**C)** (loss of) positive mood
**D)** anxiety

When recalculating correlations using these factors, the relationships remained significant, but the strengths of associations reduced [for motivation/interest cluster and perseverance: 0.439 (originally: 0.484), for motivation/interest and positive mood: 0.587 (originally: 0.680), and for motivation/interest and anxiety: 0.369 (originally: 0.461)]. This may suggest a decrease of association due to removal of theoretic ambiguity with persisting fundamental neurophysiological interrelations based on the disruption of whole neurotransmitter systems.

The identified factors are in line with previous studies proposing separate factors for negativity and anhedonia (13) and an AES-cluster of motivation and interest in a cohort of elderly individuals with mild cognitive impairment (43). In the latter study, two additional factors of “awareness” and “task completion” were proposed based on factor analysis using the AES. The strongest item loadings of the motivation/interest factor related to the items “motivation” and “initiative” which corresponds well with the proposed clinical criteria of reduced goal directed behavior and cognition in apathy and the findings of Kirsch-Darrow et al. (44) proposing a separable loss of motivation cluster.

### Apathy and Depression

After the DSM-5 criteria, a Major Depressive Disorder (MDD) can be diagnosed when the individual is experiencing five or more of a total of nine symptoms during the same 2-week period and at least one of the symptoms should be either (1) depressed mood or (2) loss of interest or pleasure. Some of these symptoms include: in- or hypersomnia, psychomotor agitation or retardation, fatigue or loss of energy, diminished ability to think or concentrate, or indecisiveness. As can be seen, even one of the cardinal features of MDD, loss of interest, but also the clinical feature of psychomotor agitation both overlap with some of the subcomponents of apathy. In fact, apathy was once considered part of the depression symptomatology. In addition, the DSM-5 includes the multidimensional syndrome of fatigue [see below, for a relationship of depression and fatigue in PD see (45)] as a possible symptom requirement for diagnosing MDD without providing further definitional information [for a critique regarding the non-strict boundaries between disorders in the DSM-5 see (46, 47) for a discussion about the usefulness of the research domain criteria (RDoC) that aims at providing an alternative framework for research into psychiatric disorders]. Taken together, the DSM diagnosis for MDD represents anything but a homogeneous categorical entity (48).

In PD, the main differentiating clinical characteristic between depression and apathy is the mood component, as it remains “neutral” in the latter and negatively affected in the former (49). While apathy is characterized by emotional indifference or lack of or decreased emotional response to positive or negative events, depression may incorporate guilt and suicidal intentions (50, 51), increased sensitivity to aversive stimuli and decreased sensitivity toward rewards. There are studies showing that apathy can indeed exist separately from depression in PD (13, 44, 52).

Regarding the interrelation of apathy and depression, we found a clear separation within the anhedonic spectrum: the (lack of) pleasure component clustered separately from the (loss of) interest component. This is in contrast to the results of Kirsch-Darrow et al. (44), who used the apathy scale (compared with the AES-scale used in the present sample) and found a combined loss of interest/pleasure factor representing a mixed apathy-depression cluster in PD patients. However, in the latter study, the Beck Depression Inventory (BDI) was used to assess depression. Interestingly, the two items representing loss of interest of the D-HADS were unable to increase model fit statistics when compared with the items representing loss of interest of the AES. Recently, the HADS has been criticized because of its lack of a good separation between symptoms of anxiety and depression and the evidence that it rather measures general distress (53). In contrast to the BDI, sadness (negative mood) is not assessed using the HADS. Moreover, the single item representing “reduced initiation” of the D-HADS was unable to contribute significantly to model fit and was therefore removed.

In general, our results indeed suggest that the motivation/interest factor (“pure apathy”; loss of interest as part of a loss of goal-directed cognition in apathy) is devoid of a hedonic component, which has relevance not only for its theoretical conceptualization but also for research and therapy. It also argues against the hypothesis that apathy might be a subcomponent of depression. Based on these findings, amotivation and loss of interest could represent a precondition for lack of emotional responses. In analogy to the gate control theory of pain, amotivation/loss of interest as a symptom that is dissociable from reduced pleasure could be understood as upstream factor necessary for the generation of emotional responses. This view could help understanding the strong interrelation between apathy and anhedonia, as for example in depression. As has been cautioned (13), using loss of interest as a symptom of anhedonia for diagnosing MDD in PD instead of a symptom of apathy could increase false positive diagnosis.

### Apathy and Anxiety

Anxiety can be defined as a future-oriented mood state associated with preparation for possible, upcoming negative events in the form of thoughts of future threat, muscle tension and avoidance (54). The current sample shows a strong positive association between apathy and anxiety. In the present sample, 26 patients showed a clinical mixture of apathy and anxiety. CFA demonstrated clearly separable clusters of loss of motivation/interest and anxiety. Regarding the co-existence of apathy and anxiety, Maillet et al. (17) could show that apathetic parkinsonian patients presented higher anxiety scores as assessed using the Lille Apathy Rating Scale and State-Trait Anxiety Inventory. A recent study investigating functional connectivity concluded that less voluntary and more automatic emotion regulation as well as an impaired ability to guide goal-directed motor actions could be characteristic in anxious PD patients (24). This, together with the involvement of diverse neurotransmitter systems in the pathophysiology of PD, provides also conceptual sense regarding the interrelation of apathy and anxiety. It is also consistent with reports showing that PD patients can suffer from anxiety and at the same time report more fatigue (45, 55).

### Apathy and Fatigue

Fatigue in PD can be subdivided into peripheral and central fatigue: whereas the first is composed of an increased loss of muscle strength after repeated contractions and can be objectively measured [sometimes also termed performance fatigability, see (22), motor fatigability: decline in peak force and cognitive fatigability: decline in reaction time or accuracy in a given task], the latter refers to a subjective state, experience or perception of (constant) exhaustion associated with a difficulty in initiating or sustaining voluntary activities that cannot be measured yet (56). Central fatigue can further be divided into physical (i.e., the amount of felt effort to complete certain activities that require skeletal muscle to generate force) and mental fatigue (i.e., the felt effort individuals have to mobilize to pay attention to certain tasks) (57). PD patients seem to suffer from greater central physical and mental fatigue compared with normal controls (58). Therefore, also in the case of apathy and fatigue, there is symptomatic overlap: the diminished goal-directed behavior domain of apathy represents a lack of effort or energy to perform everyday activities, which is also considered a clinical consequence of physical fatigue (59). Accordingly, previous studies have shown that difficulties in goal-directed behavior and cognition (apathy) are associated with increased fatigue in PD patients (57). In addition, strong associations between apathy and the multidimensional fatigue inventory (MFI)-reduced motivation domain in primary and secondary fatigue could be shown (56). However, some apathy symptoms might not be explained by a fatigue dimension (see below, outcome of CFA). In line with the described interrelation of apathy and fatigue, fatigued patients in the group of apathy represent the largest fraction in the current sample of comorbid patients (34 patients for significant apathy and severe fatigue).

Regarding the interrelation between apathy and fatigue in the current sample, the results of the CFA suggest a separation between perseverance and motivation/interest, implying that in PD, physical fatigue can be separated from amotivation/loss of interest (pure apathy). This may relate to the fact that patients suffering from fatigue usually want to do activities but are limited due to lack of energy. Fatigue is also different from depression, as it is not related to mood (and from daytime sleepiness, as fatigue does not improve after sleeping). Furthermore, our results provide evidence for a separation between physical fatigue and depression. Not carefully distinguishing perseverance from MDD and PD could similarly lead to false positive diagnosis.

### Clinical Implications

Support for the separability of apathy, fatigue and depression in PD has broad theoretical and clinical implications. It suggests different neural mechanisms may underlie apathy, fatigue and depression. It implies that clinicians should screen for apathy, fatigue and depression to appropriately treat patients. Briefly, there are yet no specific treatment options for apathy or fatigue in Parkinson's disease. There is evidence that rasagiline and levodopa infusion therapy for fatigue and rivastigmine for apathy might be effective treatment options (27, 60, 61). Congruently in our data there was a negative correlation between the AES score and the total dose of dopamine agonists. Most antidepressant therapy (62), but also some dopamine agonists (63), show partial efficacy in patients with PD, the latter potentially aiming at the motivational component of the heterogeneous depressive syndrome. A factor-based scoring of the AES, FSS, and HADS that disentangles symptoms related to apathy, depression and fatigue as a subsidiary line of evidence may aid in patient care.

## Conclusion

To our knowledge, this is one of the first studies trying to separate apathy from fatigue, depression and anxiety using CFA in a large sample of PD patients. In accordance with previous studies, our data of 337 PD patients suggest that apathy, depression, anxiety and fatigue are prevalent syndromes in PD. However, comparing prevalence estimates between studies is often hampered as they show a substantial variation. This heterogeneity may be grounded on the current limited methodological possibilities which rely largely on self-report questionnaires of different scales applied to non-uniform PD patient cohorts and using different cut-off thresholds. Therefore, it is difficult to assess whether there is a diagnosis bias or if these constructs share common etiologic mechanisms. Our data suggest that apathy, depression, anxiety and fatigue are strongly interrelated making the development of a clearer theoretical and neural mechanistic separation a corner stone for future individualized treatment. In line with previous studies on the interrelation of apathy and fatigue, fatigued patients in the group of apathy represent the largest fraction in the current sample of comorbid patients. Using confirmatory factor analysis, apathy could be separated from depression, anxiety and fatigue: Pure apathy, defined as loss of motivation and interest, could be dissociated from physical fatigue, loss of positive affect (anhedonia) and anxiety. This argues against the hypothesis that apathy might be a subcomponent of depression. A clearer conceptual understanding of apathy, fatigue and depression in Parkinson's disease may aid in therapeutic management and research strategies. Further studies are needed to more conclusively clarify the relationship between apathy, fatigue, depression, and anxiety but also cognition and motor status.

## Limitations

The current study has several limitations. First, from a methodological view, a larger sample could have been split to first conduct an exploratory factor analysis and to test the resulting empirically informed clusters using confirmatory factor analysis on the second half of the data. However, the statistical power analysis revealed that to detect a small-to-medium effect size, the current sample was just large enough. Therefore, we refrained from splitting the data. Second, the HADS has been shown to measure general distress rather than anxiety or depression specifically (53) apart from lacking a sadness-dimension. Third, the FSS scale lacks a clear definition of fatigue making it difficult to attribute item clusters to the various subcomponents of fatigue.The FSS also has a clear focus on physical fatigue, relativizing the emergence of the described item-cluster in this study. Based on the fact that it was no aim of the study to deconstruct fatigue and given the importance of the FSS in the clinical assessment of fatigue in PD, the choice of scale can be legitimized.

## Data Availability Statement

The data analyzed in this study is subject to the following licenses/restrictions: Institutional health related data. Requests to access these datasets should be directed to Christian Ineichen, christian.ineichen@usz.ch.

## Ethics Statement

The studies involving human participants were reviewed and approved by the cantonal ethics committee Zurich. The patients provided their written informed consent to participate in this study.

## Author Contributions

CI performed the analyses and confirms that he has final responsibility for the decision to submit for publication. HB-V provided clinical expertise for statistical modeling. CI and HB-V wrote the paper. All authors contributed to the article and approved the submitted version.

## Conflict of Interest

The authors declare that the research was conducted in the absence of any commercial or financial relationships that could be construed as a potential conflict of interest.

## Publisher's Note

All claims expressed in this article are solely those of the authors and do not necessarily represent those of their affiliated organizations, or those of the publisher, the editors and the reviewers. Any product that may be evaluated in this article, or claim that may be made by its manufacturer, is not guaranteed or endorsed by the publisher.
