# Supplementary material for: Deconstructing Apathy in Parkinson's Disease: Challenges in Isolating Core Components of Apathy From Depression, Anxiety, and Fatigue
Source: Front Neurol. 2021 Aug 26;12:720921. doi: 10.3389/fneur.2021.720921 (PMC8427284; doi:10.3389/fneur.2021.720921)
Supplement: Supplementary file 1 [file Table_1.docx]

Supplementary Material

# Supplementary Table S1

Theoretical 4-factor Model:

1. Proposed apathy cluster (items):
   1. AES_StartingThingsImportant
   2. AES_InterestedInThings
   3. AES_GetThingsDone
   4. AES_Effort
   5. AES_GetJobDone
   6. AES_TimeSpending
   7. AES_MotivatorNeeded
   8. AES_LessConcerned
   9. AES_Excited
   10. AES_NewExperiences
   11. AES_InterestedLearning
   12. AES_Vitality
   13. AES_GettingThingsDoneDaily
   14. AES_Initiative
   15. AES_Motivated
   16. FSS_LessMotivated
   17. D-HADS_FeelRestrained
   18. D-HADS_InterestLost
2. Proposed depression cluster (items):
   1. D-HADS_FutureIsBright
   2. D-HADS_HappyAsBefore
   3. D-HADS_Happy
   4. D-HADS_CanLaugh
3. Proposed anxiety cluster (items):
   1. A-HADS_Worrisome
   2. A-HADS_Premonition
   3. A-HADS_WorryingGutFeeling
   4. A-HADS_Panic
4. Proposed fatigue cluster (items):

- Physical fatigue:
  1. FSS_TiredQuickly
  2. FSS_ProductivityAffected
  3. FSS_Tiresome
  4. FSS_ProductivityInhibited
- Disease burden:
  1. FSS_DutiesAffected
  2. FSS_IsMostAffectingProblem
  3. FSS_DutiesInhibited
